# Proteogenomic Characterization of Monocyclic Aromatic Hydrocarbon Degradation Pathways in the Aniline-Degrading Bacterium *Burkholderia* sp. K24

**DOI:** 10.1371/journal.pone.0154233

**Published:** 2016-04-28

**Authors:** Sang-Yeop Lee, Gun-Hwa Kim, Sung Ho Yun, Chi-Won Choi, Yoon-Sun Yi, Jonghyun Kim, Young-Ho Chung, Edmond Changkyun Park, Seung Il Kim

**Affiliations:** 1 Drug & Disease Target Team, Korea Basic Science Institute, 169–148 Gwahak-ro, Yuseong-gu, Daejeon, Republic of Korea; 2 Bio-Analysis Science, University of Science & Technology, 217 Gajeong-ro, Yuseong-gu, Daejeon, Republic of Korea; 3 Department of Food Science and Technology, Chungnam National University, Daejeon, 305–764, Republic of Korea; Wageningen University, NETHERLANDS

## Abstract

*Burkholderia* sp. K24, formerly known as *Acinetobacter lwoffii* K24, is a soil bacterium capable of utilizing aniline as its sole carbon and nitrogen source. Genomic sequence analysis revealed that this bacterium possesses putative gene clusters for biodegradation of various monocyclic aromatic hydrocarbons (MAHs), including benzene, toluene, and xylene (BTX), as well as aniline. We verified the proposed MAH biodegradation pathways by dioxygenase activity assays, RT-PCR, and LC/MS-based quantitative proteomic analyses. This proteogenomic approach revealed four independent degradation pathways, all converging into the citric acid cycle. Aniline and *p*-hydroxybenzoate degradation pathways converged into the β-ketoadipate pathway. Benzoate and toluene were degraded through the benzoyl-CoA degradation pathway. The xylene isomers, i.e., *o*-, *m*-, and *p*-xylene, were degraded via the extradiol cleavage pathways. Salicylate was degraded through the gentisate degradation pathway. Our results show that *Burkholderia* sp. K24 possesses versatile biodegradation pathways, which may be employed for efficient bioremediation of aniline and BTX.

## Introduction

Aniline (aminobenzene) is a toxic organic compound, used as a precursor for dyes, herbicides, plastics, paints, rubber additives, pesticides, and pharmaceuticals [1]. Because aniline is widely used in industrial products, its accumulation and toxicity have become an ecological problem. Aniline is considered toxic by inhalation of vapor, ingestion, or percutaneous absorption. It is a carcinogen and mutagen, and belongs to Category 3 on the International Agency for Research on Cancer (IARC) list [2].

Aniline biodegradation genes and major metabolic enzymes were reported for several aniline-biodegrading bacterial strains. *Pseudomonas* [3], *Acinetobacter* [4], *Rhodococcus* [5], *Frateuria* [6], and *Delftia* [7] are all capable of degrading and utilizing aniline as a carbon and nitrogen source. However, until now, whole genome sequence data of aniline-biodegrading bacteria was insufficient for understanding the aniline degradation mechanisms and their metabolic characterization.

Recently, we reported a draft genome of the aniline-degrading bacterium *Burkholderia* sp. K24 [8]. *Burkholderia* sp. K24 was previously known as *Acinetobacter lwoffii* K24. In a previous study, we found that *Acinetobacter lwoffii* K24 used two intradiol cleavage pathway (β-ketoadipate pathway) genes for aniline degradation [9], and we confirmed their activity by gel-based proteomic approaches [10]. We also confirmed the presence of an alternative branch of the β-ketoadipate pathway for *p*-hydroxybenzoate degradation of *Acinetobacter lwoffii* K24 [11].

In this study, we performed a comprehensive genomic and proteomic analysis, i.e., proteogenomic analysis, to comprehensively evaluate biodegradation activity of *Burkholderia* sp. K24. Proteogenomic approaches are useful tools for the identification and elucidation of bacterial metabolic pathways because putative biodegradation pathways initially predicted by genomic analysis can then be verified by a proteomic analysis. Specifically, quantitative proteomics can indicate which pathways play major metabolic roles under specific culture conditions. *Burkholderia* sp. K24 possesses additional biodegradation activities for monocyclic aromatic hydrocarbons (MAHs), including aniline, benzoate, *p*-hydroxybenzoate, salicylate, benzene, toluene, and xylene analogues. Our study proposes *Burkholderia* sp. K24 degradation pathways of MAHs, including benzene, toluene, and xylene (BTX). BTX is toxic or carcinogenic to humans, and many BTX-degrading bacteria and their genome sequence have been reported [12]. However, until now, no genome of bacteria degrading both aniline and BTX has been reported. To the best of our knowledge, this is the first proteogenomic report on an aniline-degrading bacterium that also degrades other mono-aromatic hydrocarbons, including BTX.

## Materials and Methods

### Bacterial cultivation

*Burkholderia* sp. K24 cells were pre-cultured in potassium phosphate buffer (pH 6.25) containing 3.4 mM MgSO_4_, 0.3 mM FeSO_4_, 0.2 mM CaCO_3_, 10 mM NH_4_Cl, and 10 mM sodium succinate, and then transferred to one of the following solutions: succinate (10 mM), benzoate (10 mM), *p*-hydroxybenzoate (5 mM), salicylic acid (2-hydroxybenzoic acid) (5 mM), toluene (methylbenzene) (400 ppm), benzene (400 ppm), or *o*-, *m*-, *p*-xylene (100 ppm). The cells were then cultured aerobically at 30°C. In the case of aniline (1000 ppm), the same medium as the basal medium was used, except that it was not supplemented with NH_4_Cl. Cultured bacteria were harvested in the late exponential phase for enzyme activity assays and proteomic analysis. Harvested bacteria were suspended in 20 mM Tris-HCl buffer (pH 8.0) and disrupted twice in a French pressure cell (SPCH-10, Standard Fluid Power Ltd, UK) at 20,000 psi. Supernatants (crude cell extracts) were collected by centrifugation (15,000 × g, 45 min) and used in enzyme activity assays and proteomic analyses.

### Activity assays of catechol dioxygenase, protocatechuate dioxygenase, and gentisate dioxygenase enzymes

Catechol 1,2-dioxygenase activity was measured spectrometrically at 260 nm. Increase of *cis*,*cis*-muconate concentration was used as a measure of enzyme activity [13]. One unit of enzyme activity is defined as the amount of enzyme required to produce 1μmol of *cis*,*cis*-muconate per min. Catechol 2,3-dioxygenase activity was measured spectrometrically at 375 nm. Increase of 2-hydroxymuconic semialdehyde concentration was an indicator of enzyme activity [13]. Protocatechuate 3,4-dioxygenase and protocatechuate 4,5-dioxygenase activities were measured at 290 and 410 nm, respectively. Increase of β-carboxymuconate and 2-hydroxy-4-carboxymuconic semialdehyde concentrations was used to assess enzyme activity [13]. Gentisate 1,2-dioxygenase activity was measured spectrometrically at 330 nm, according to a reported method [14]. One unit of enzyme activity is defined as the amount of enzyme required to produce 1 μmol of maleylpyruvate per min. Absorbance was measured using a UV spectrometer (Beckman Coulter Proteome Lab DU800, USA)

### Sodium dodecyl sulfate-polyacrylamide gel electrophoresis and in-gel tryptic digestion

Crude protein mixtures were fractionated by sodium dodecyl sulfate (12%)-polyacrylamide gel electrophoresis (SDS-PAGE). Tryptic digestion for MS/MS analysis was performed as described previously [15]. SDS-polyacrylamide gels were then divided into ten fragments according to molecular weight. After reduction with 10 mM dithiothreitol and alkylation of cysteines with 55 mM iodoacetamide, the gel fragments were digested with trypsin (Promega, Madison, WI, USA) for 16 h at 37–8°C. The digested peptides were extracted with extraction solution [50 mM ammonium bicarbonate, 50% acetonitrile, and 5% trifluoroacetic acid (TFA)]. For liquid chromatography-tandem mass spectrometry (LC-MS/MS) analysis, the samples were then dissolved in 0.5% TFA.

### LC-MS/MS analysis

Tryptic peptide samples (10 μL) were concentrated using a MGU-30 C18 trapping column (LC Packings, Amsterdam, the Netherlands), eluted, and directed onto a C18 reverse-phase column (10 cm × 5 mm I.D.; Proxeon Biosystems, Odense, Denmark) at a flow rate of 120 nL/min. Peptide mixtures were eluted with a gradient of 0–65% acetonitrile for 70 min. All MS and MS/MS spectra were acquired with a LTQ-Velos ESI Ion Trap mass spectrometer (Thermo Scientific, Germany). Three MS/MS scans of the most abundant precursor ions with the dynamic exclusion feature enabled were selected from each full MS (*m*/*z* range 400–2000) scan. Protein identification was performed using MASCOT v2.4 (Matrix Science, Inc., Boston, MA). The protein sequence database of *Burkholderia* sp. K24 was downloaded from NCBI and used for MS/MS data analysis. Oxidation of methionine, carbamidomethylation of cysteines, two missed trypsin cleavages, peptide tolerance of 0.8 Da, and fragment mass tolerance of 0.8 Da comprised search parameters. The exponentially modified protein abundance index (emPAI) was generated using MASCOT, with mol% calculated according to emPAI values [16]. MS/MS analysis was performed at least three times for each sample. MS/MS data were filtered assuming a 1% false discovery rate (FDR).

### Bioinformatics

Sequences of 16S rRNA genes from 33 stains of *Burkholderia*, *Pseudomonas*, and *Acinetobacter* species were obtained from the SILVA database and used for phylogenetic tree construction [17]. Sequence alignments were analyzed, and phylogenetic tree was generated using MEGA 6.0 [18]. Aromatic compound biodegradation pathway genes were predicted using KEGG BlastKOALA and MetaCyc [19]. Degrading genes unidentified in KEGG BlastKOALA were putatively identified by sequence homology analyses with orthologous degrading gene sequences (E-value < 1e-20) using BlastP. Subcellular localization of all of the hypothetical proteins was predicted by Cello v2.5 [20].

### RT-PCR

Equal volumes of RNAprotect Bacterial Reagent (Qiagen) were added to bacterial cultures to stabilize RNA. To analyze gene expression, total RNA was extracted from *Burkholderia* sp. K24 using the RNeasy Mini Kit (Qiagen), and the cDNA library was synthesized with the QuantiTect Reverse Transcription Kit (Qiagen) using 500 ng of total RNA, and stored at -20°C. 16S rRNA of *Burkholderia* sp. K24 was used as a reference to estimate gene expression levels. Real-Time PCR (RT-PCR) primers used were as follows: 16S rRNA, 5′-GGAGCCATAACACAGGTGCT-3′ and 5′-TCACCGGCAGTCT CCTTAGA-3′; *amtB* (KBK24-0108070), 5′-TAGATCAGCGTCGTCAGCAC-3′ and 5′-AGCACAAGC TCGGTTACGAC-3′; and *amtB* (KBK24-0129725), 5′-TGATCTTGTCGATGGTCTGC-3′ and 5′-CG TCGAATATCCCGTTCCT-3′. Because *Burkholderia* sp. K24 has two amtB genes (KBK24_0108070 and KBK_0129725), two different primer sets were used. Other RT-PCR primers for confirmation of biodegradation pathways were listed in S1 Table. RT-PCR (Roche, LightCycler 480 with software version 1.5.1.62) was performed with the following cycling conditions: pre-heating (one cycle), 95°C for 5 min with 4.4°C/s ramp rate; amplification (45 cycles), 95°C for 10 s with 4.4°C/s ramp rate, 60°C for 20 s with 2.2°C/s ramp rate, 72°C for 10 s with 4.4°C/s ramp rate; melting curve analysis and cooling, 95°C for 5s with 4.4°C/s ramp rate, 65°C for 1 min with 2.2°C/s ramp rate, and five acquisitions per 1°C with 0.11°C/s ramp rate.

## Results and Discussion

### Screening of *Burkholderia* sp. K24 biodegradation activities

MAH biodegradation activities of *Burkholderia* sp. K24 were screened after bacterial culturing in minimal media with single MAHs as sole carbon sources (S1 Fig). In our study, *Burkholderia* sp. K24 was able to use nine MAHs as sole carbon sources and grew well, to OD 0.3–1.0. To predict which metabolic pathways of *Burkholderia* sp. K24 were required for MAH utilization, enzyme activity assays of five major dioxygenases were performed using ten different exponential phase cultures [21]. Table 1 shows dioxygenase induction according to culture conditions. Based on these results, we predicted that the β-ketoadipate pathway, extradiol cleavage pathway, and gentisate pathways were induced for the utilization of aniline, *p*-hydroxybenzoate, toluene, xylenes, and salicylate. However, no dioxygenase activities were detected during growth on benzoate and benzene, suggesting that *Burkholderia* sp. K24 uses other biodegradation pathways for these MAHs.

**Table 1:** Dioxygenase activities of *Burkholderia* sp. K24 for monocyclic aromatic compounds.

Substrate	Specific Enzyme Activity (U/mg)				
	CD1,2 ^a)^	CD2,3 ^a)^	PCD3,4 ^a)^	PCD4,5 ^a)^	GD1,2 ^a)^
Succinate	-	-	-	-	-
Aniline	0.484	-	-	-	-
*p*-Hydroxybenzoate	-	-	2.248	-	-
Salicylate	-	-	-	-	0.053
Benzoate	-	-	-	-	-
Benzene	-	-	-	-	-
Toluene	-	0.105	-	-	-
*o*-Xylene	-	1.238	-	-	-
*m*-Xylene	-	2.059	-	-	-
*p*-Xylene	-	4.930	-	-	-

^a)^ CD1,2; catechol 1,2-dioxygenase, CD2,3; catechol 2,3-dioxygenase, PCD3,4; protocatechuate 3,4-dioxygenase, PCD4,5; protocatechuate 4,5-dioxygenase, GD1,2; gentisate 1,2-dioxygenase, -; enzyme activities were not detected.

### Genomic analysis of *Burkholderia* sp. K24

Draft genome sequence of *Burkholderia* sp. K24 was reported in a previous study [8]. Phylogenetic analysis of 16S rRNA sequences revealed that *Burkholderia* sp. K24 belongs to the non-pathogenic *Burkholderia* group (S2 Fig). The group comprises *Burkholderia phytofirmans* PsJN, *Burkholderia xenoborans* LB400, *Burkholderia phenolirup-tirix* BR3459*a*, *Burkholderia pymatum* STM815, and *Burkholderia kuruiensis* M130. These micro-organisms can be potentially used as agricultural biocontrol agents or mutualists [22]. Among the annotated 7033 genes of *Burkholderia* sp. K24, 1880 genes are categorized into five major biological functions in the KEGG pathway database and 183 genes were included into one of five major biological functions, xenobiotic biodegradation and metabolism in the genome of *Burkholderia* sp. K24 (data not shown).

### Genomic prediction of aniline-, *p*-hydroxybenzoate-, benzoate-, and xylene-degrading pathways of *Burkholderia* sp. K24

**Fig 1:**
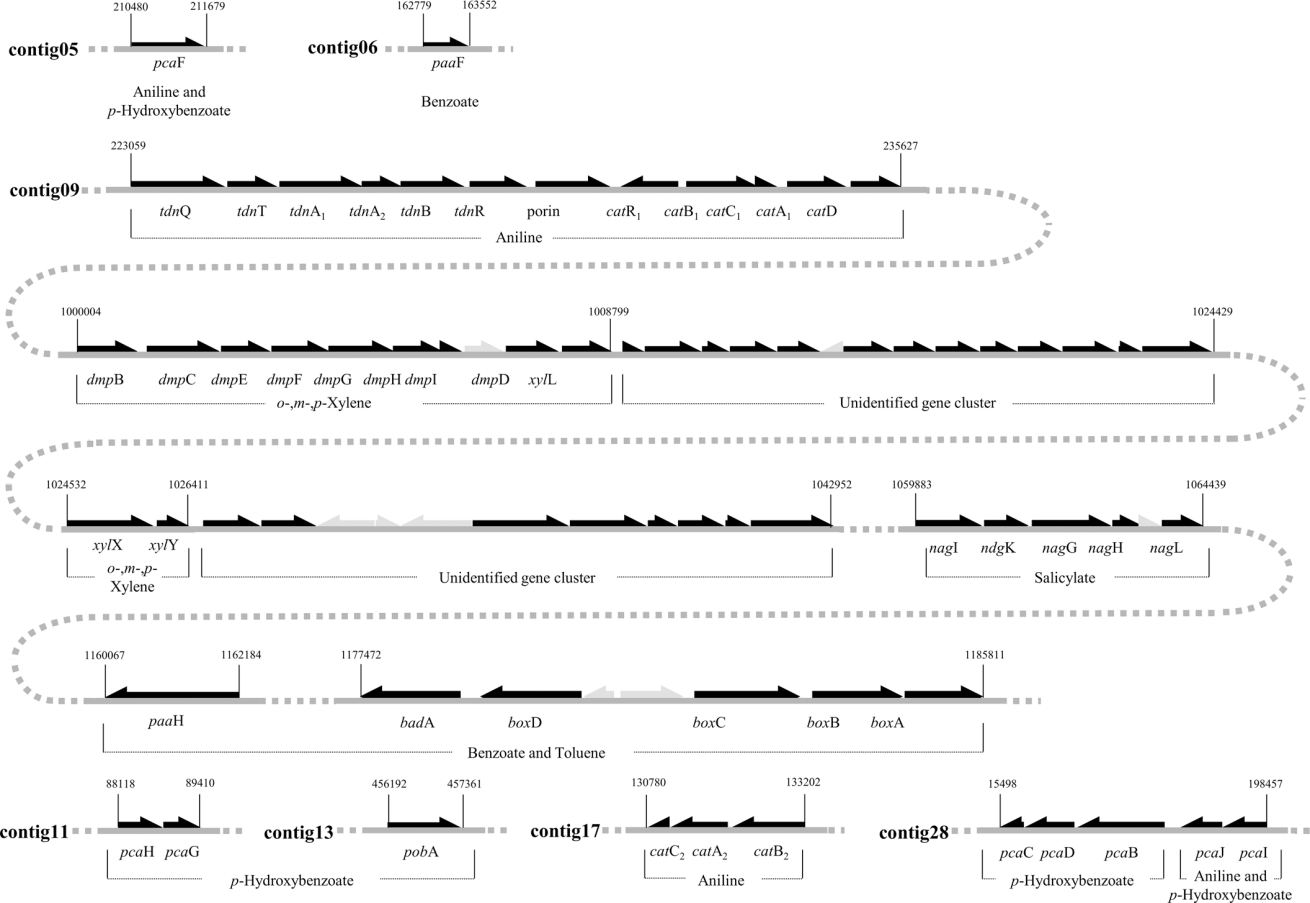
Organization of gene clusters involved in biodegradation of monocyclic hydrocarbons aniline, *p*-hydroxybenzoate, salicylate, toluene, benzoate, and xylene analogues in the *Burkholderia* sp. K24 genome.

Many identified putative biodegradation genes of *Burkholderia* sp. K24 were found concentrated in contig 9, with the remaining biodegradation genes scattered in the remaining six contigs (Fig 1). Aniline degradation genes are located in four contigs (5, 9, 17, and 28). The 223059–235627 region of contig 9 covers 12 genes, which include the aniline oxygenase complex (*tdnQTA*_*1*_*A*_*2*_*BR*-Porin), and the *cat*_1_ gene cluster (*catR*_*1*_*B*_*1*_*C*_*1*_*A*_*1*_*D*_*1*_). Another *cat* gene cluster, *cat*_2_ (*catC*_*2*_*A*_*2*_*B*_*2*_), was found in the 130780–133202 region of contig 17 (Fig 1). The two *cat* gene clusters were identified in previous studies [23, 24]. However, the aniline oxygenase complex is herein identified for the first time. The genomic analysis revealed that *Burkholderia* sp. K24 degrades aniline (aminobenzene) through an intradiol cleavage pathway (β-ketoadipate pathway). However, genes of the latter stages of the β-ketoadipate pathway (*catIJF*) were not found in the aniline degradation gene cluster in contig 9. The genes encoding the latter stages of the β-ketoadipate pathway and *p*-hydroxybenzoate degradation pathways were scattered in four contigs (5, 11, 13, and 28). In conclusion, this genomic analysis suggested that *Burkholderia* sp. K24 employs the β-ketoadipate pathway for the utilization of aniline and *p*-hydroxybenzoate. In addition, comparative sequence analysis revealed a high degree of homology between aniline and *p*-hydroxybenzoate degradation genes of *Burkholderia* sp. K24 and the genes of *Frateuria* sp. ANA-18 and *Burkholderia fungorum* ATCC BAA-463 [25, 26] (S2 Table).

Contrary to the majority of β-ketoadipate pathway-utilizing bacteria, the benzoate oxygenase gene complex (*benABCD*) was not detected in *Burkholderia* sp. K24, suggesting that this strain does not use the β-ketoadipate pathway for benzoate degradation. Instead, we found another benzoate oxidation (*box*) gene cluster, in the 1177472–1185811 region of contig 9 (Fig 1). This cluster contained benzoate degradation genes for benzoate oxidation via benzoyl-CoA. Presumably, benzoate is degraded to 2,3-dihydro-2,3-dihydroxybenzoyl-CoA via benzoyl-CoA by benzoate-CoA ligase and benzoyl-CoA dioxygenase components (BadA-BoxAB). Following this, 2,3-dihydro-2,3-dihydroxybenzoyl-CoA would be converted to acetyl-CoA and succinyl-CoA by benzoyl-CoA-dihydrodiol lyase and aldehyde dehydrogenase (Ben BoxCD-PaaHF-PcaF). Similar biodegradation pathways were found in *Burkholderia fungorum* ATCC BAA-463, *Azoarcus evansii* and *Burkholderia xenovorans* LB400 [25].

The eight genes involved in the degradation pathways of methyl-catechol to pyruvate and propanoyl-CoA were clustered in the 1000004–1008799 region of contig 9 (Fig 1). This cluster enables β-ketoadipate extradiol degradation through catechol 2,3-dioxygenase. This gene cluster was assumed to be responsible for the degradation of xylenes because the catechol 2,3-dioxygenase activity was induced in *Burkholderia* sp. K24 cultured in the presence of xylenes, as assessed in the dioxygenase activity assays (Table 1), and no other extradiol cleavage pathway was identified in the genome. However, genes of the early stages of the xylene degradation pathway were not identified by the comparative genome analysis. These genes share the highest sequence homology with genes of the phenanthrene-degrading bacterium *Burkholderia* sp. HB-1 [27] (S2 Table). Five salicylate degradation genes were also located in the 1059883–1064439 region of contig 9 (Fig 1). This suggested that *Burkholderia* sp. K24 is able to degrade salicylate to gentisate using the salicylate 5-hydroxylase complex (NagG and NagH), and the products of that reaction are further catabolized by the gentisate degradation pathway.

Using sequence analysis, we were unable to establish the pathways for toluene and benzene degradation. Catechol 2,3-dioxygenase activity was detected in *Burkholderia* sp. K24 cultures grown in the presence of toluene, but the enzyme activity was low. We therefore proceeded to use the proteomic analysis to verify whether the extradiol pathway is the major or alternative toluene degradation pathway in this bacterium.

### Proteomic analysis of *Burkholderia* sp. K24 MAH degradation pathways

We used proteomic analysis to verify the predictions of the genomic analysis and identify other possible degradation pathways from protein induction patterns. We employed a LC/MS-based shotgun method for quantitative proteomics of *Burkholderia* sp. K24 cultured under ten different conditions (Table 2).

**Table 2:** Summary of proteomic results of *Burkholderia* sp. K24 cultured in different aromatic hydrocarbons.

Predicted localization (CELLO v 2.5)	Succinate	Aniline	*p*-Hydroxy benzoate	Benzoate	Salicylate	*o*-Xylene	*m*-Xylene	*p*-Xylene	Toluene	Benzene
Cytoplasmic	1243	983	1016	996	1206	1086	1098	1088	1109	1382
Periplasmic	272	249	252	224	257	242	240	249	259	311
Outer Membrane	43	46	50	46	41	36	36	43	35	49
Inner Membrane	81	40	48	57	51	59	48	65	63	111
Extracellular	36	33	40	35	32	28	30	29	28	38
Total	1675	1351	1406	1358	1587	1451	1452	1474	1494	1891

Between 1351 and 1891 proteins were identified and quantified according to emPAI analysis, respectively, from each bacterial culture condition. A total of 2594 proteins were identified, covering about 38% of the genome. About 14–119 proteins were exclusively induced under each culture condition. On the other hand, 740 proteins were commonly induced under all culture conditions. Spearman coefficients for 740 proteins commonly induced in the ten bacterial cultures were 0.71–0.99 (data not shown). This suggested that these proteins were similarly induced under all of the culture conditions and may thus play essential or overlapping physiological functions. Quantification of each protein was performed by mol% calculation on the basis of emPAI values, and abundant proteins were identified in each proteome set.

A DNA-binding protein, GroEL and GroES, and the universal stress protein UspA were identified as abundant proteins. Another abundant protein was ribosomal proteins, which was significantly variable depending on culture conditions (data not shown). Specifically, ribosomal proteins were up-regulated in succinate cultures, compared with the monocyclic aromatic cultures (1.52–1.68-fold), suggesting that protein synthesis and cell growth were more robust in the succinate cultures.

### Proteomic analysis of *Burkholderia* sp. K24 aniline-, p-hydroxybenzoate-, and benzoate-degrading pathways

In our earlier proteomic studies, we confirmed the induction of the β-ketoadipate pathway during growth in the presence of aniline and *p*-hydroxybenzoate [11, 28]. However, at that time, we had only obtained fragmentary sequence information for 43 major proteins identified in those studies [10, 28]. On the other hand, the current LC/MS-based proteomic analysis yielded a more comprehensive proteomic dataset. Here, 1351 proteins of *Burkholderia* sp. K24 cultured in aniline-containing medium were identified and quantified. Enzymes of the initial stages of the aniline degradation pathway (TdnQABT and CatABCD) were selectively induced under these conditions (Table 3). However, enzymes of the latter stages of the β-ketoadipate pathway (PcaIJF) were induced in both aniline- and *p*-hydroxybenzoate-containing cultures, suggesting that these enzymes were required for utilization of both of these aromatic compounds (Table 3). Enzymes of the initial stages of the *p*-hydroxybenzoate degradation pathway (PobA, PcaGH) were exclusively induced in *p*-hydroxybenzoate cultures (Table 3). This study allowed us to complete the β-ketoadipate pathway of *Burkholderia* sp. K24 and confirmed a selective induction of the two branches of the β-ketoadipate pathway, depending on the availability of aniline and *p*-hydroxybenzoate, respectively. Additionally, quantitative analysis revealed enzymes that play major roles in biodegradation, e.g., two catechol 1,2-dioxygenases, CatA_1_ and CatA_2_. Our data revealed CatA_1_ is 5.6-fold highly induced, suggesting that CatA_1_ is the major enzyme catalyzing the cleavage of catechol.

**Table 3:** Summary of differential proteomic expression of *Burkholderia* sp. K24 cultured in monocyclic aromatic hydrocarbons.

Substrates	Locus tag	Gene Name	Product	Protein abundance (mol%)									
				Suc	Ani	p-Hydro	Benzo	Sal	*o*-Xyl	*m*-Xyl	*p*-Xyl	Tol	Benze
Aniline	KBK24_0116295	tdnQ	glutamine synthetase	-	0.100	-	-	0.022	-	-	-	-	-
	KBK24_0116305	tdnA^1^	large subunit of dioxygenase	-	0.080	-	-	0.008	-	-	-	-	-
	KBK24_0116306	tdnA_2_	small subunit of dioxygenase	-	0.086	-	-	0.008	-	-	-	-	-
	KBK24_0116315	tdnB	aniline dioxygenase reductase	-	0.021	-	-	-	-	-	-	-	-
	KBK24_0116296	tdnT	glutamine amidotransferase	-	0.065	-	-	-	-	-	-	-	-
	KBK24_0116345	catA^1^	catechol 1,2-dioxygenase	-	0.856	-	-	0.032	-	-	-	-	0.001
	KBK24_0116335	catB^1^	muconate cycloisomerase	-	0.075	-	-	-	-	-	-	-	0.001
	KBK24_0116340	catC^1^	muconolactone delta-isomerase	-	0.136	-	-	-	-	-	-	-	-
	KBK24_0135040	catA_2_	catechol 1,2-dioxygenase	-	0.152	0.002	-	0.013	-	-	0.006	0.005	0.005
	KBK24_0135045	catB_2_	muconate cycloisomerase	-	0.066	-	-	0.005	-	-	-	-	-
	KBK24_0135035	catC_2_	muconolactone delta-isomerase	-	0.071	-	-	0.022	-	-	-	-	0.002
	KBK24_0116350	catD	3-oxoadipate enol-lactonase	-	0.048	-	-	-	-	-	-	-	-
Aniline and *p*-Hydroxybenzoate	KBK24_0138210	pcaI	3-oxoadipate CoA-transferase, α subunit	0.010	0.075	0.063	0.032	0.023	-	0.070	-	0.025	0.003
	KBK24_0138205	pcaJ	3-oxoadipate CoA-transferase, β subunit	-	0.033	0.032	0.009	0.007	-	0.065	-	-	0.002
	KBK24_0109905	pcaF	β-ketoadipyl CoA thiolase	0.019	0.129	0.052	0.011	0.058	0.015	0.192	0.033	0.033	0.024
*p*-Hydroxybenzoate	KBK24_0131110	pobA	*p*-hydroxybenzoate 3-monooxygenase	-	0.002	1.347	0.059	-	-	-	-	-	-
	KBK24_0125685	pcaG	protocatechuate 3,4-dioxygenase, α subunit	-	-	1.102	-	-	-	-	-	-	-
	KBK24_0125680	pcaH	protocatechuate 3,4-dioxygenase, β subunit	-	-	0.748	-	-	-	-	0.007	-	-
	KBK24_0138200	pcaB	3-carboxy-cis,cis-muconate cycloisomerase	-	0.006	0.010	0.001	0.012	-	0.030	0.021	-	-
	KBK24_0138190	pcaC	4—carboxymuconolactone decarboxylase	0.015	0.045	0.031	0.024	0.039	0.037	0.068	0.061	0.049	0.049
	KBK24_0138195	pcaD	3-oxoadipate enol-lactonase	0.028	0.089	0.070	0.033	0.059	0.039	0.088	0.120	0.041	0.109
Benzoate and Toluene	KBK24_0120745	badA	benzoate-CoA ligase	-	-	-	0.031	-	-	-	-	0.048	-
	KBK24_0120775	boxA	benzoyl-CoA 2,3-dioxygenase component A	-	-	-	0.010	-	-	-	-	0.034	-
	KBK24_0120770	boxB	benzoyl-CoA 2,3-dioxygenase component B	-	-	-	0.229	-	0.004	0.009	0.007	0.125	0.005
	KBK24_0120765	boxC	benzoyl-CoA-dihydrodiol lyase	0.004	-	0.002	0.169	-	0.006	0.005	0.005	0.153	0.005
	KBK24_0120750	boxD	aldehyde dehydrogenase	-	-	-	0.085	-	-	-	-	0.116	-
	KBK24_0113935	paaF	enoyl-CoA hydratase	0.063	0.036	0.014	0.089	0.094	0.067	0.088	0.255	0.109	0.285
	KBK24_0120670	paaH	3-hydroxyacyl-CoA dehydrogenase	-	-	0.001	0.007	-	-	0.002	0.002	0.009	-
*o*-,*m*-,*p*-Xylene	KBK24_0120105	xylX	benzoate/toluate 1,2-dioxygenase alpha subunit	-	-	-	-	-	-	0.004	0.007	0.021	0.005
	KBK24_0120110	xylY	benzoate/toluate 1,2-dioxygenase beta subunit	-	-	-	-	-	0.064	0.162	0.277	0.016	0.306
	KBK24_0120035	xylL	1,6-dihydroxycyclohexa-2,4-diene-1-carboxylate dehydrogenase	-	-	-	-	-	0.067	0.075	0.088	0.014	-
	KBK24_0119990	dmpB	catechol 2,3-dioxygenase	-	-	-	-	-	0.575	1.040	0.349	0.043	-
	KBK24_0119995	dmpC	2-hydroxymuconate-semialdehyde hydrolase	-	-	-	-	-	0.116	0.088	0.067	0.010	-
	KBK24_0120030	dmpD	aminomuconate-semialdehyde	-	-	-	-	-	0.009	0.053	0.084	0.017	-
	KBK24_0120020	dmpI	4-oxalocrotonate tautomerase	-	-	-	-	-	0.492	0.673	0.615	0.032	-
	KBK24_0120015	dmpH	4-oxalocrotonate decarboxylase	-	-	-	-	-	0.087	0.096	0.264	0.030	-
	KBK24_0120000	dmpE	2-keto-4-pentenoate hydratase	-	-	-	-	-	0.106	0.111	0.186	0.016	-
	KBK24_0120010	dmpG	4-hyroxy-2-oxovalerate aldolase	-	-	-	-	-	0.084	0.215	0.097	0.047	-
	KBK24_0120005	dmpF	acetaldehyde dehydrogenase	-	-	-	-	-	0.217	0.425	0.160	0.057	-
Salicylate	KBK24_0120295	nagG	salicylate 5-hydroxylase large subunit	-	-	-	-	0.059	-	-	-	-	-
	KBK24_0120300	nagH	salicylate 5-hydroxylase small subunit	-	-	-	0.005	0.103	0.028	-	-	-	-
	KBK24_0120285	nagI	gentisate 1,2-dioxygenase	0.008	-	-	0.003	0.166	0.014	-	-	-	-
	KBK24_0120310	nagL	maleylpyruvate isomerase	-	-	-	-	0.010	-	-	-	-	-
	KBK24_0120290	nagK	fumarylpyruvate hydrolase	-	-	-	0.003	0.097	-	-	-	-	-
Citrate cycle^1^	KBK24_0126135	cs	type II citrate synthase	0.102	0.087	0.079	0.091	0.174	0.123	0.090	0.077	0.115	0.066
	KBK24_0126195	aco	aconitate hydratase	0.162	0.638	0.373	0.413	0.278	0.196	0.099	0.165	0.270	0.159
	KBK24_0117400	idh1	isocitrate dehydrogenase	0.035	0.029	0.029	0.033	0.061	0.057	0.038	0.038	0.062	0.027
	KBK24_0129934	sucA	2-oxoglutarate dehydrogenase	0.097	0.018	0.018	0.023	0.077	0.020	0.020	0.039	0.076	0.029
	KBK24_0129935	dlst	dihydrolipoamide succinyltransferase	0.093	0.097	0.075	0.066	0.085	0.053	0.051	0.058	0.068	0.047
	KBK24_0116610	sucC	succinyl-CoA synthetase subunit beta	0.283	0.166	0.128	0.160	0.150	0.286	0.268	0.250	0.333	0.285
	KBK24_0126145	sdhB	succinate dehydrogenase	0.034	0.059	0.033	0.057	0.036	0.027	0.026	0.073	0.038	0.062
	KBK24_0135320	fumA	fumarate hydratase	0.061	0.009	0.008	0.012	0.076	0.029	0.024	0.047	0.060	0.035
	KBK24_0126170	mdh	malate dehydrogenase	0.570	1.360	0.606	0.705	0.840	0.740	0.791	0.631	0.752	0.827
Un-identified proteins	KBK24_0120040		anthranilate 1,2-dioxygenase	-	-	-	-	-	0.062	0.269	0.234	0.061	-
	KBK24_0120045		Rieske (2Fe-2S) protein	-	-	-	-	-	0.008	0.053	0.034	-	-
	KBK24_0120050		DSBA oxidoreductase	-	-	-	-	-	0.096	0.399	0.509	0.011	0.577
	KBK24_0120055		NADPH:quinone reductase	-	-	-	-	0.004	0.025	0.167	0.083	0.017	-
	KBK24_0120060		naphthalene 1,2-dioxygenase	-	-	-	-	-	0.392	0.369	0.269	0.236	0.303
	KBK24_0120070		flavin-dependent oxidoreductase	-	-	-	-	-	0.435	0.345	0.223	0.316	0.241
	KBK24_0120075		alpha/beta hydrolase	-	-	-	-	-	0.192	0.326	0.766	0.171	0.987
	KBK24_0120080		hypothetical protein	-	-	-	-	-	0.057	0.066	0.056	0.085	0.045
	KBK24_0120085		aldolase	0.004	-	-	-	0.005	1.054	0.734	0.426	0.753	0.468
	KBK24_0120090		(2Fe-2S)-binding protein	-	-	-	-	-	0.108	0.080	0.051	0.074	0.039
	KBK24_0120095		hypothetical protein	-	-	-	-	-	0.069	0.138	0.181	0.051	0.184
	KBK24_0120100		hypothetical protein	-	-	-	-	-	0.021	0.032	0.055	0.051	0.044
	KBK24_0120115		4-hydroxythreonine-4-phosphate dehydrogenase	-	-	-	-	-	0.075	0.060	0.070	0.085	0.060
	KBK24_0120120		dihydrodipicolinate synthetase	-	-	-	-	-	0.071	0.050	0.039	0.041	0.028
	KBK24_0120160		naphthalene 1,2-dioxygenase	-	-	-	-	-	0.159	0.065	0.059	0.156	0.048
	KBK24_0120165		aromatic-ring-hydroxylating dioxygenase	-	-	-	-	-	0.285	0.585	0.941	0.182	1.124
	KBK24_0120170		2,3-dihydroxy-2,3-dihydrophenylpropionate dehydrogenase	-	-	-	-	-	0.204	0.302	0.538	0.396	0.648
	KBK24_0120180		ATP:cob(I)alamin adenosyltransferase	-	-	-	-	0.013	0.244	0.291	0.659	0.297	0.845
	KBK24_0120185		aldehyde dehydrogenase	-	-	-	-	-	0.218	0.188	0.155	0.347	0.143

Suc, Succinate; Ani, Aniline; *p*-Hydro, *p*-Hyroxybenzoate; Benzo, Benzoate; Sal, Salicylate; *o*-Xyl, *o*-Xylene; *m*-Xyl, *m*-Xylene; *p*-Xyl, *p*-Xylene; Tol, Toluene; Benze, Benzene

^1^ The average range of proteome amount of TCA cycle enzymes were calculated from 0.042 ~ 0.782 of mol %

**Fig 2:**
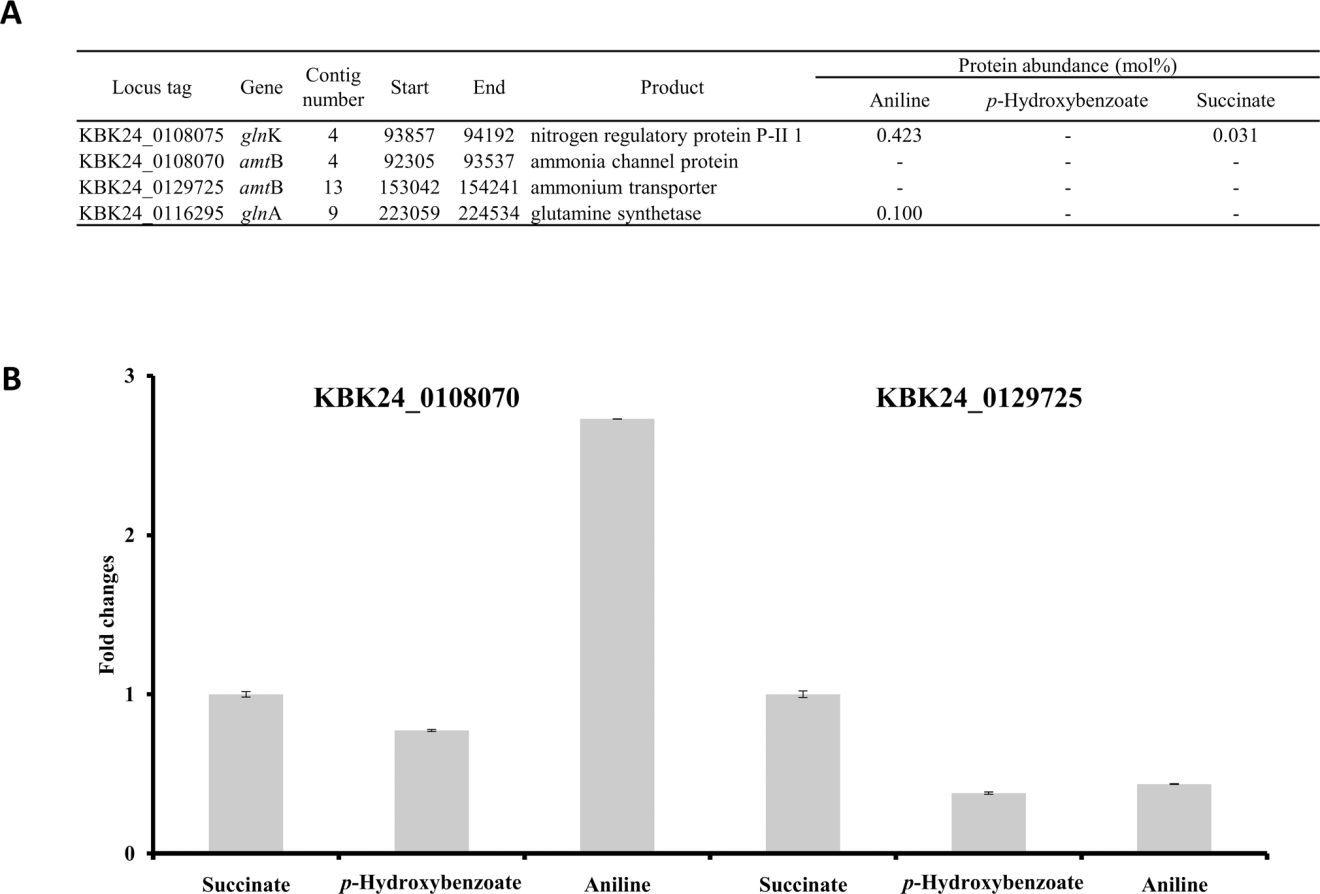
Proteomic evidence of ammonium assimilation enzymes (A), and RT-PCR data for the two *amtB* genes (KBK24_0108070 and KBK24_0129725) (B) from different *Burkholderia* sp. K24 culture conditions.

Because aniline was used as the sole carbon and nitrogen source, the ammonia released from the aniline amino group was most likely assimilated by *Burkholderia* sp. K24 to provide nitrogen. Proteomic analysis showed that several nitrogen assimilating proteins were indeed induced in aniline-containing cultures (data not shown). Two glutamine synthetases, specifically, GlnK (nitrogen regulatory protein P-II 1, KBK24_0108075), were noticeably induced by aniline (Fig 2A). *E*. *coli* GlnK plays a role in ammonium influx, together with the ammonium transporter AmtB [29]. In the case of *Burkholderia* sp. K24, two AmtB proteins (KBK24_0108070 and KBK24_0129725) were identified but were not detected in our proteome analysis (Fig 2B). Since AmtBs are membrane proteins, they were not detectable in the soluble fraction of the proteome. Nevertheless, RT-PCR showed that one *amtB* gene (KBK24_0108070) was significantly induced by aniline (Fig 2B). This suggested that GlnK and AmtB play major roles in ammonium assimilation in *Burkholderia* sp. K24.

Based on our genomic analysis, we predicted that *Burkholderia* sp. K24 employed the benzoate oxidation pathway operating via benzoyl CoA, instead of the β-ketoadipate pathway. Proteomics revealed that seven enzymes involved in benzoyl-CoA degradation pathways were selectively up-regulated, confirming this pathway as the major degradation pathway (Table 3). However, enzymes of the *p*-hydroxybenzoate branch of the β-ketoadipate pathway were also detected in benzoate-containing medium. Induction of the *p*-hydroxybenzoate branch of the β-ketoadipate pathway was not tightly controlled, and proteins from the *p*-hydroxybenzoate branch of the β-ketoadipate pathway were detected under all culture conditions used in this study (Table 3).

### Proteomic analysis of *Burkholderia* sp. K24 BTX and salicylate biodegradation

Our genomic analysis did not unequivocally verify the presence of pathways for BTX degradation. The results of dioxygenase activity assays and the proteomic analysis revealed that the extradiol cleavage pathway was strongly induced when *Burkholderia* sp. K24 was cultured in the presence of the three xylene isomers (Tables 1 and 3). These results suggested that this pathway plays a major role in the degradation of *o*-, *m*-, and *p*-xylene in *Burkholderia* sp. K24. When bacteria were cultured in the presence of toluene, only weak catechol 2,3-dioxygenase activity was induced and enzymes for the extradiol cleavage pathway were up-regulated (Tables 1 and 3). However, strong induction of the benzoyl CoA pathway was observed in toluene-containing cultures (Table 3). Therefore, we propose that two pathways are involved in toluene degradation, even though the benzoyl CoA pathway was previously thought to be the major pathway. We also detected an uncharacterized gene cluster, which was exclusively and strongly induced in the presence of xylenes, toluene, and benzene (Fig 1 and Table 3). This gene cluster contained a putative dioxygenase, an oxidoreductase, and an aldolase. We did not identify homologous gene clusters in other MAH-biodegrading bacteria using comparative gene analysis. Even though more functional evidence is required, the results of our proteomic analysis support the possibility that the gene cluster is involved in BTX degradation. Salicylate degradation genes predicted by the genomic analysis were also confirmed by the proteomic analysis when *Burkholderia* sp. K24 was cultured in the presence of salicylate (Table 3).

### RT-PCR analysis of major biodegradation genes of *Burkholderia* sp. K24

To confirm the proteomic results, the transcriptional levels of major genes belong to each biodegradation pathway were assay by RT-PCR. Eight genes (*cat*A_1_, *cat*A_2_, *pca*G, *pca*H, *box*B, *dmp*B, *nag*I, and *nag*H) were assayed in nine culture conditions (S3 Fig). Two *cat genes* (*cat*A_1_ and *cat*A_2_) and *pca*GH were dominantly induced in aniline and *p*-hydroxybenzoate, respectively. *Box*B was exclusively induced in benzoate and toluene, which is consistent with the proteomic result (Table 3). *Dmp*B is highly induced in only in three xylene analogues among the nine cultures, suggesting catechol 2,3-dioxygenase pathways are major degradation pathways for xylene analogues.

### Proteomic analysis of the TCA cycle of *Burkholderia* sp. K24 utilizing MAHs

**Fig 3:**
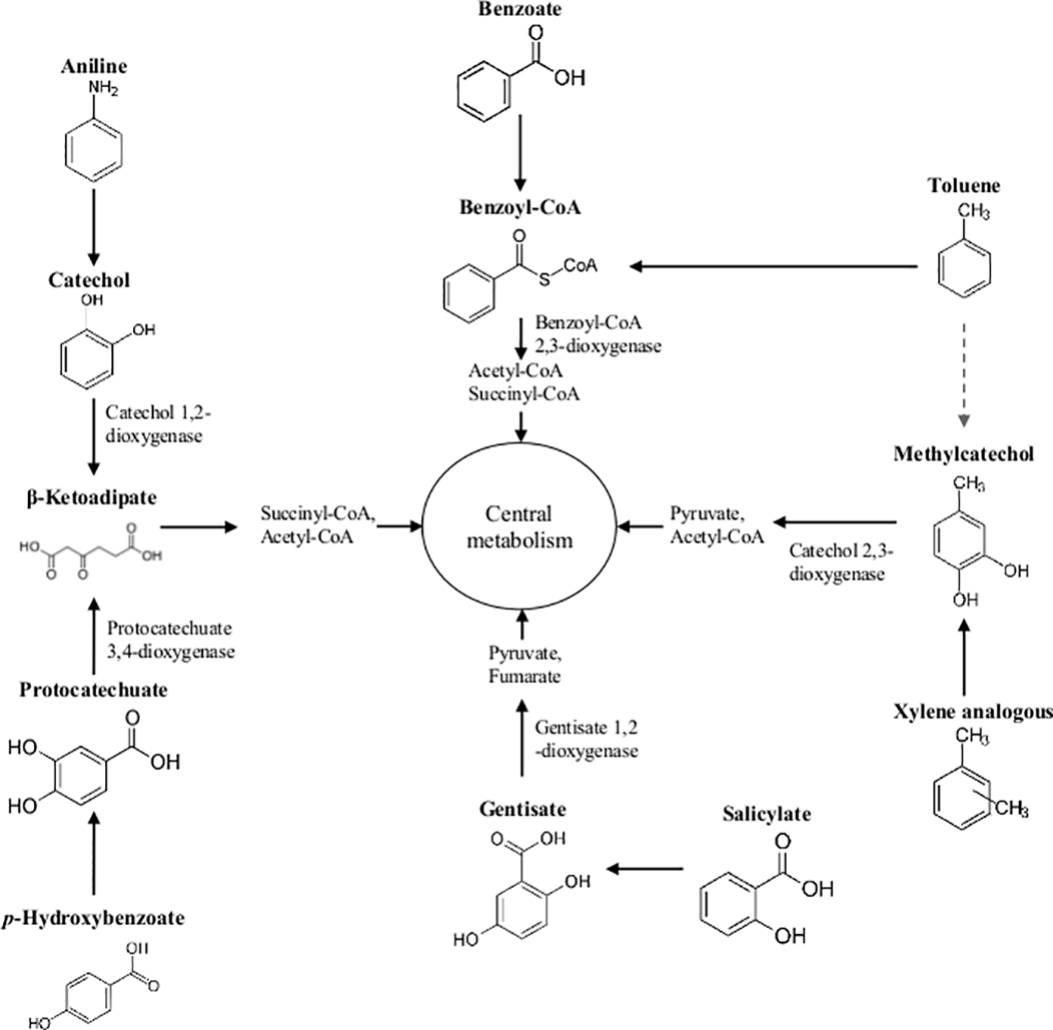
Overview of monocyclic hydrocarbon biodegradation pathways in *Burkholderia* sp. K24.

According to our proteogenomic analysis of MAH biodegradation pathways, the resultant metabolites acetyl-CoA, succinyl-CoA, fumarate, and pyruvate flow into the tricarboxylic acid (TCA) cycle to generate other cellular building blocks or energy (Fig 3). Therefore, we investigated the proteomic patterns of TCA enzymes of *Burkholderia* sp. K24 cultured in the presence of succinate and MAHs. Regardless of the culture conditions, four TCA cycle enzymes (Citrate synthase, Aconitase, Succinyl-CoA synthetase, and Malate dehydrogenase) were highly induced (Table 3). It is not surprising that the enzymes, which utilize acetyl-CoA and succinyl-CoA as substrates (Aconitase, Succinyl-CoA synthetase, and Malate dehydrogenase), would be highly induced in the presence of various MAHs and in succinate-containing media. This suggested that the TCA cycle plays a major role in catabolism and anabolisms of *Burkholderia* sp. K24.

## Conclusions

In our previous studies, we reported *Burkholderia* sp. K24 to be the first bacterium using the β-ketoadipate pathway for biodegradation of aniline and *p*-hydroxybenzoate. Genomic analysis of *Burkholderia* sp. K24 revealed various biodegradation pathways for other MAHs. Proteogenomic analysis was performed to obtain an integrated overview of the MAH biodegradation pathways and their induction characteristics. The analysis confirmed versatile *Burkholderia* sp. K24 biodegradation pathways and enzymes, which can be used for bioremediation. In our future studies, we will analyze how can *Burkholderia* sp. K24 utilize MAHs under mixed culture conditions and which MAH degradation pathways have priority for biodegradation

## Supporting Information

S1 Fig Cultivation of *Burkholderia* sp. K24 with different monocyclic aromatic hydrocarbons. Bacteria were harvested after the late exponential phase and used in enzyme activity assays and proteomic analysis.
(PDF)

S2 Fig Phylogenic tree of *Burkholderia* sp. analyzed with MEGA 6.0. (PDF)

S3 Fig Result of RT-PCR of MAH degradation genes of *Burkholderia* sp. K24. (PDF)

S1 Table RT-PCR primers for biodegradation pathways of *Burkholderia* sp. K24. (DOCX)

S2 Table Homology analysis of MAH degradation genes of *Burkholderia* sp. K24. (DOCX)
