# Correction: Proteogenomic Characterization of Monocyclic Aromatic Hydrocarbon Degradation Pathways in the Aniline-degrading Bacterium *Burkholderia* sp. K24

**DOI:** 10.1371/journal.pone.0157201

**Published:** 2016-06-03

**Authors:** Sang-Yeop Lee, Gun-Hwa Kim, Sung Ho Yun, Chi-Won Choi, Yoon-Sun Yi, Jonghyun Kim, Young-Ho Chung, Edmond Changkyun Park, Seung Il Kim

The following information is missing from the Funding section: This study was supported by the Korea Basic Science Institute research program (D35402), the Bio-Synergy Research Project (NRF- 2014M3A9C4066461) of the Ministry of Science, ICT and Future Planning through the National Research Foundation, Basic Science Research Program through the National Research Foundation of Korea funded by the Ministry of Education of Korea (2014R1A6A3A0 1059331).
