# Supplementary figures and images for: Proteogenomic Characterization of Monocyclic Aromatic Hydrocarbon Degradation Pathways in the Aniline-Degrading Bacterium Burkholderia sp. K24
Source: PLoS One. 2016 Apr 28;11(4):e0154233. doi: 10.1371/journal.pone.0154233 (PMC4849787; doi:10.1371/journal.pone.0154233)

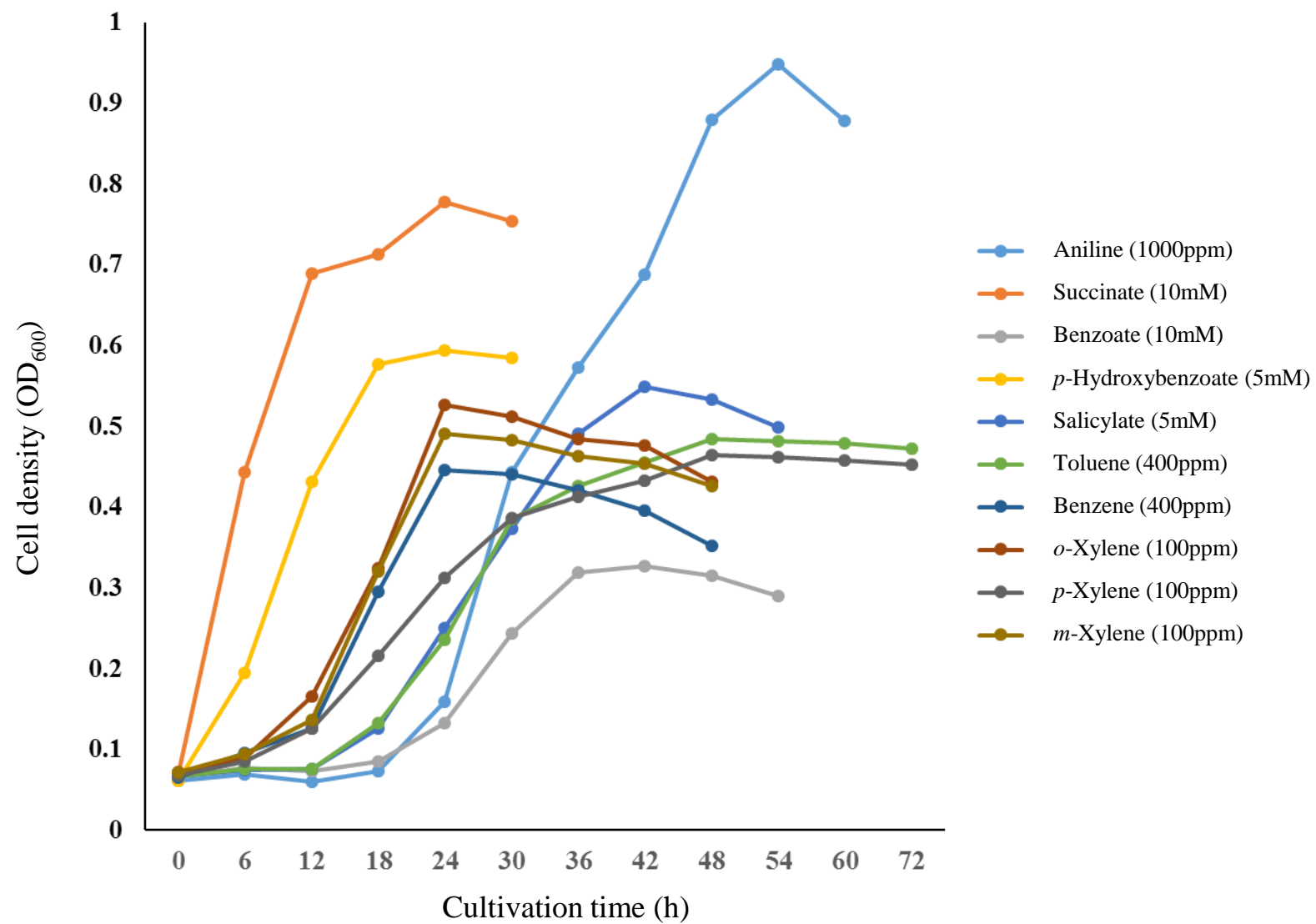

Supplement: S1 Fig — Bacteria were harvested after the late exponential phase and used in enzyme activity assays and proteomic analysis. (PDF) [file pone.0154233.s001.pdf]

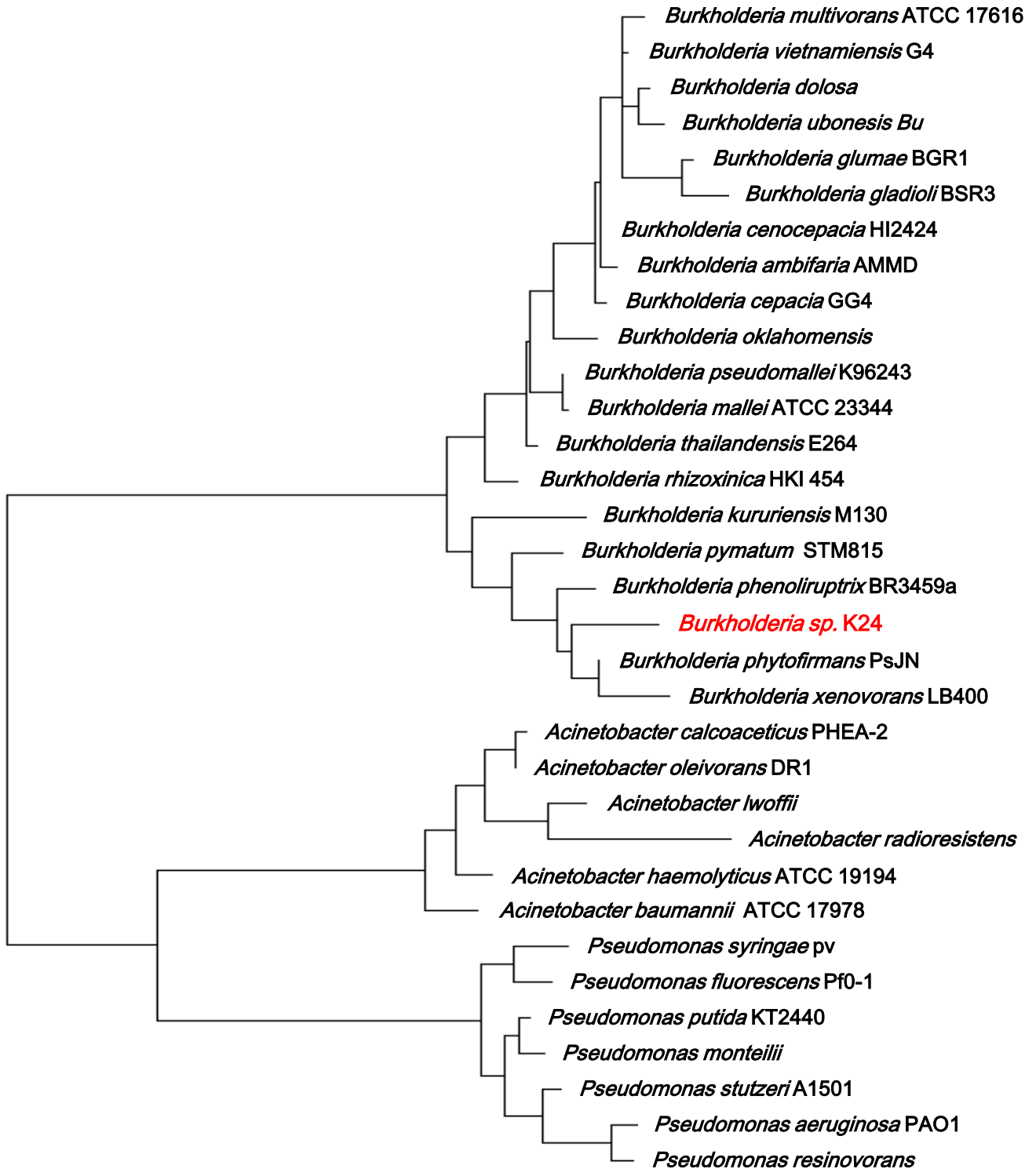

0.02

Supplement: S2 Fig — (PDF) [file pone.0154233.s002.pdf]

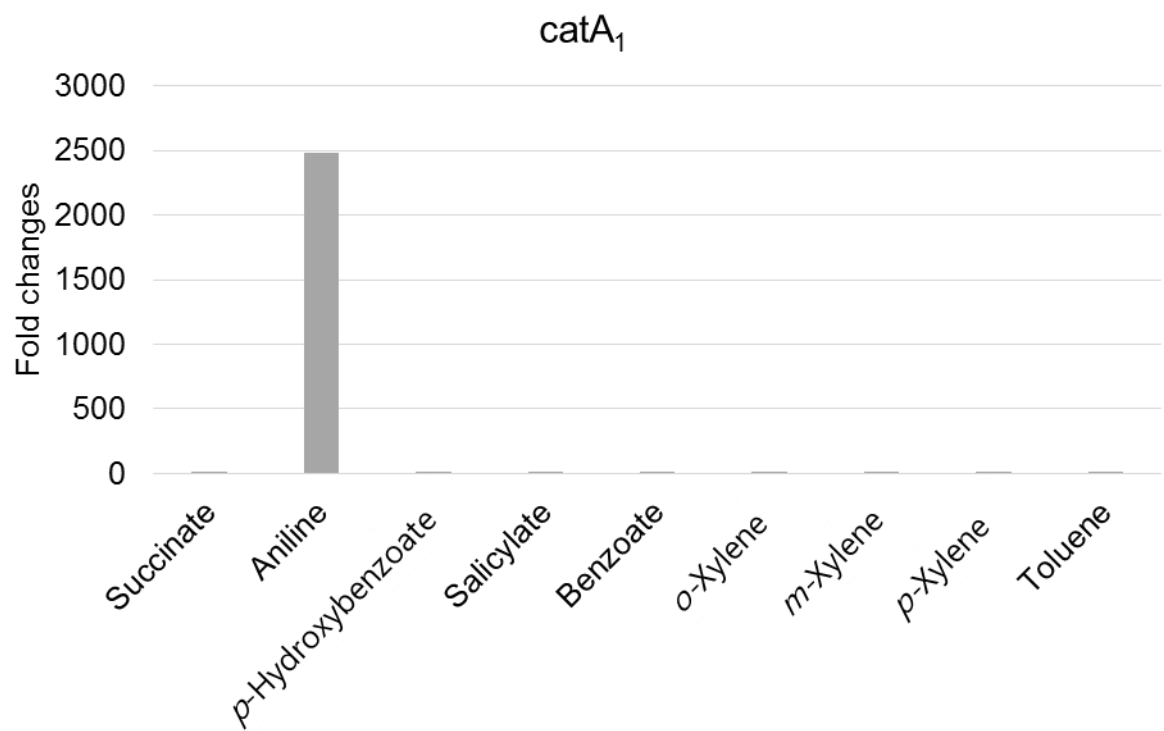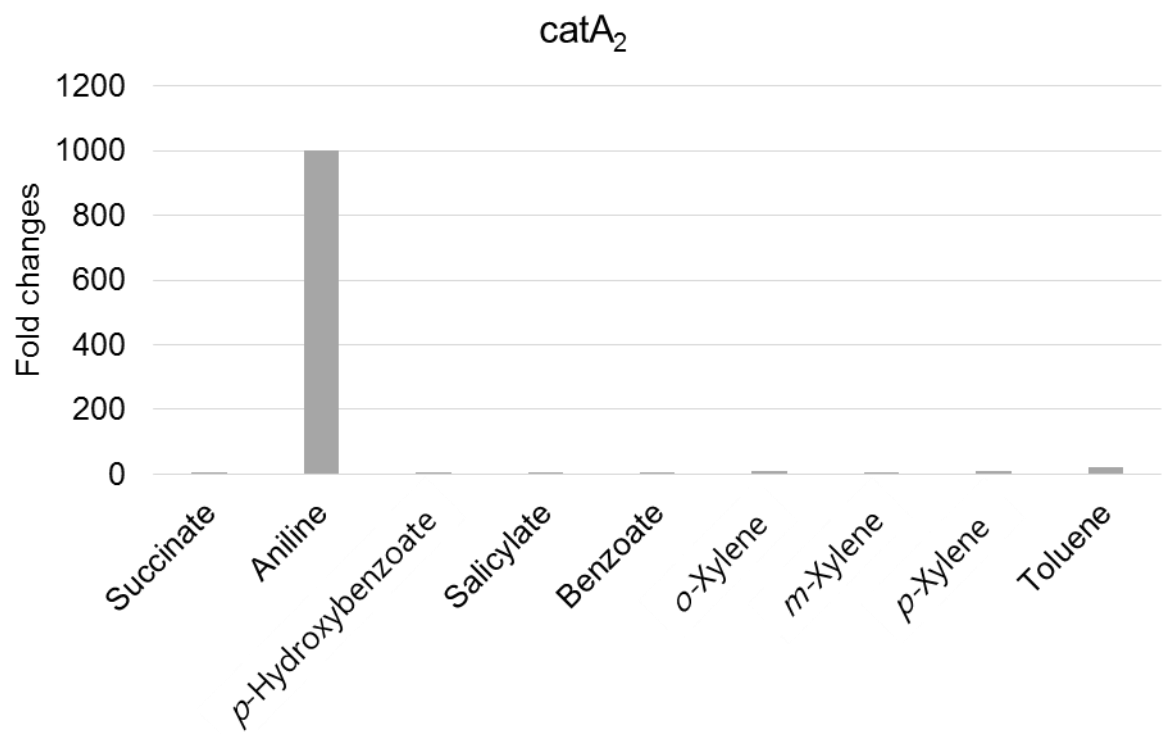

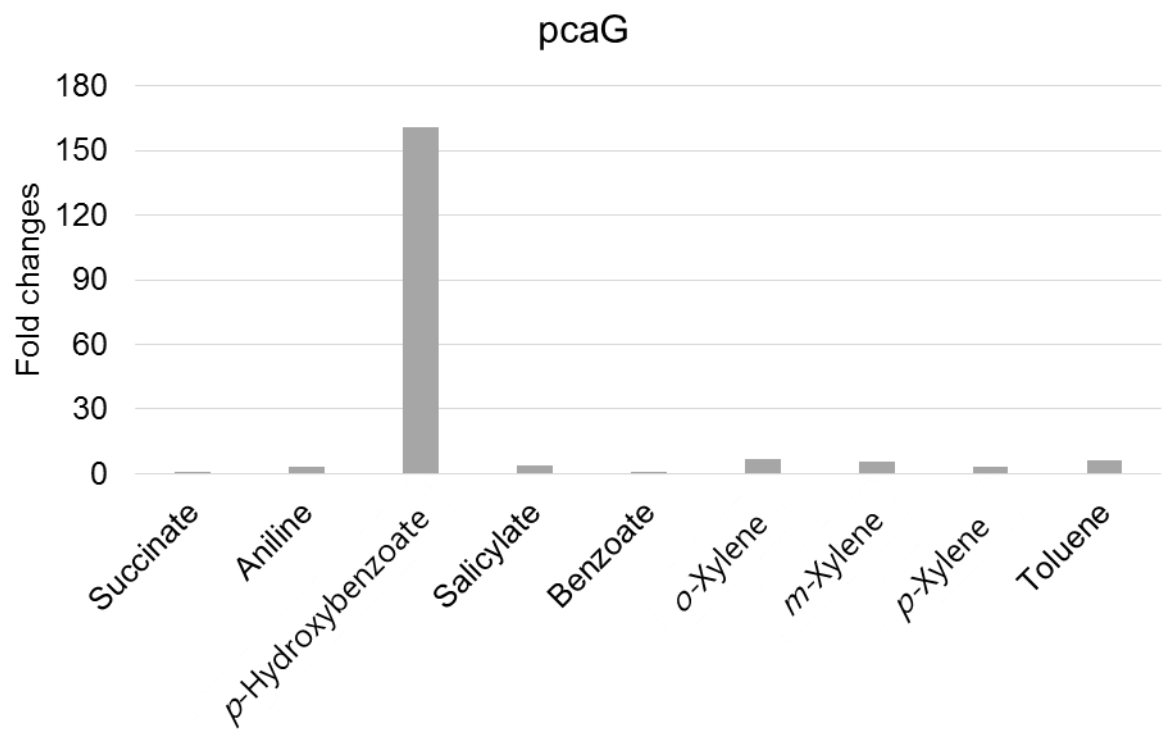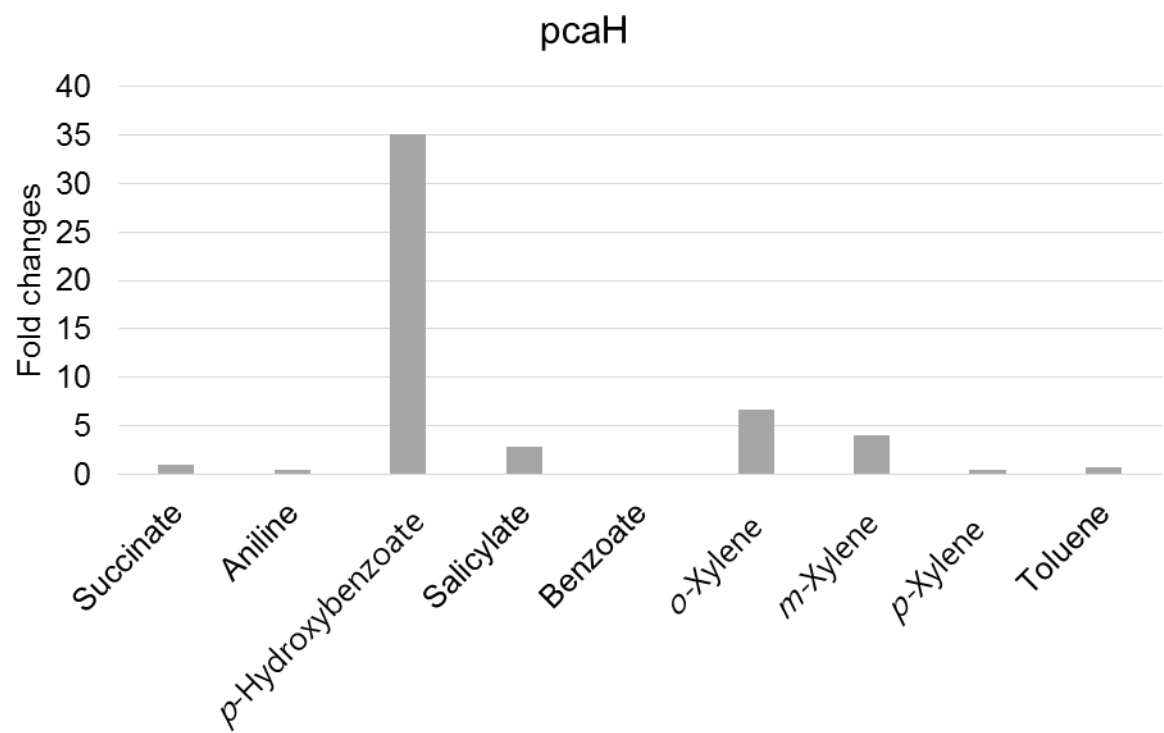

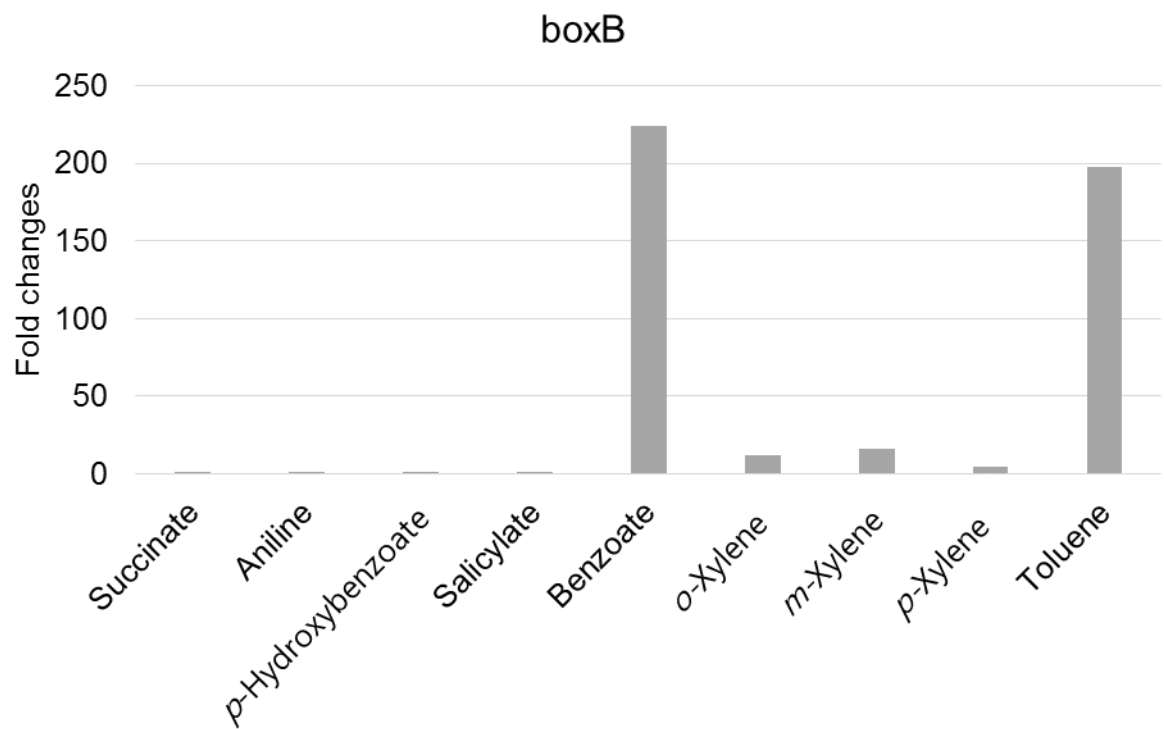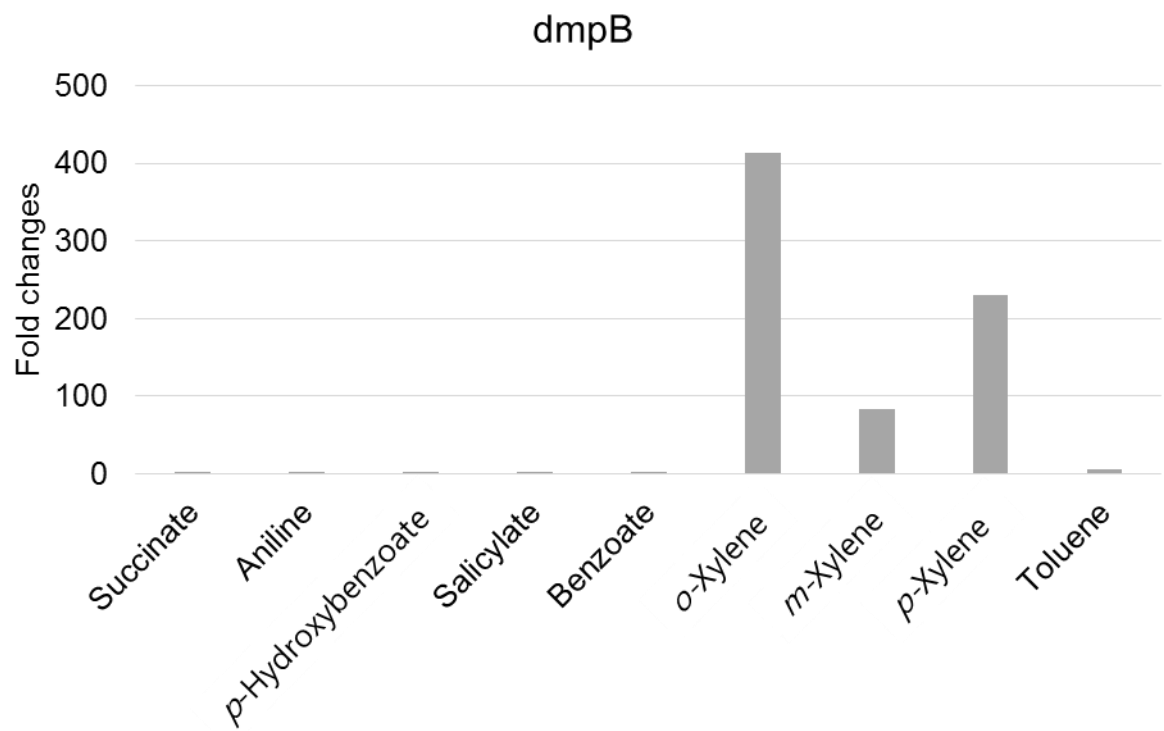

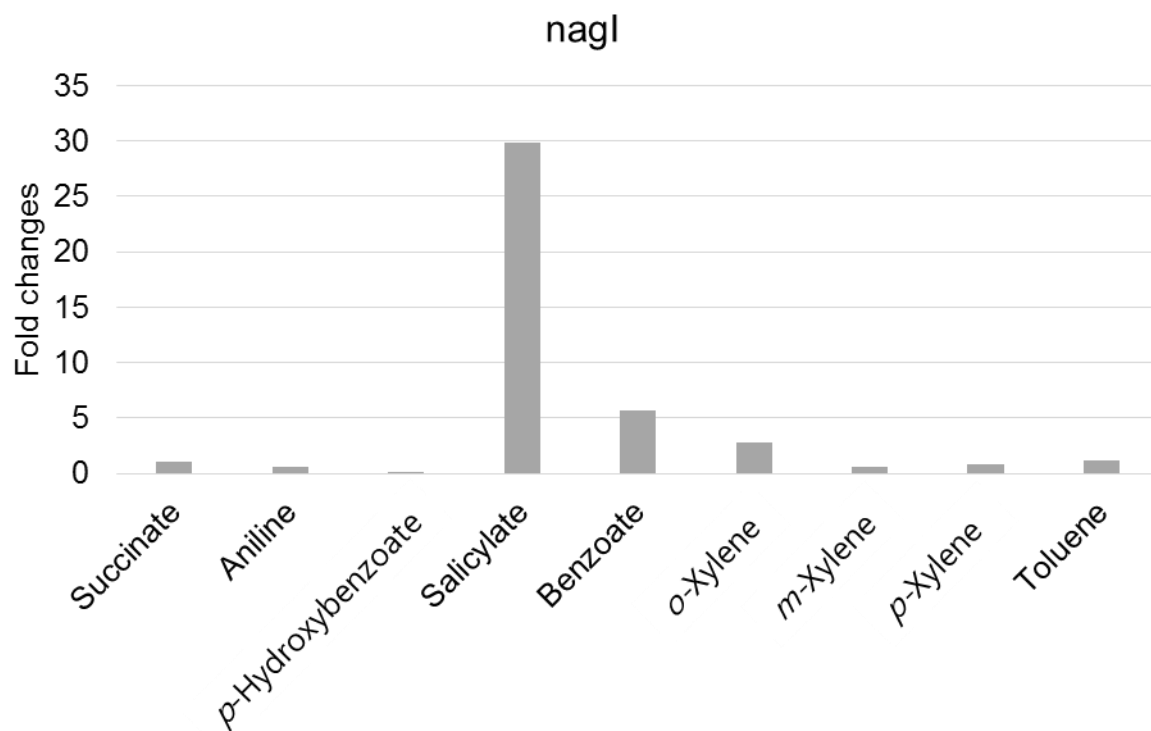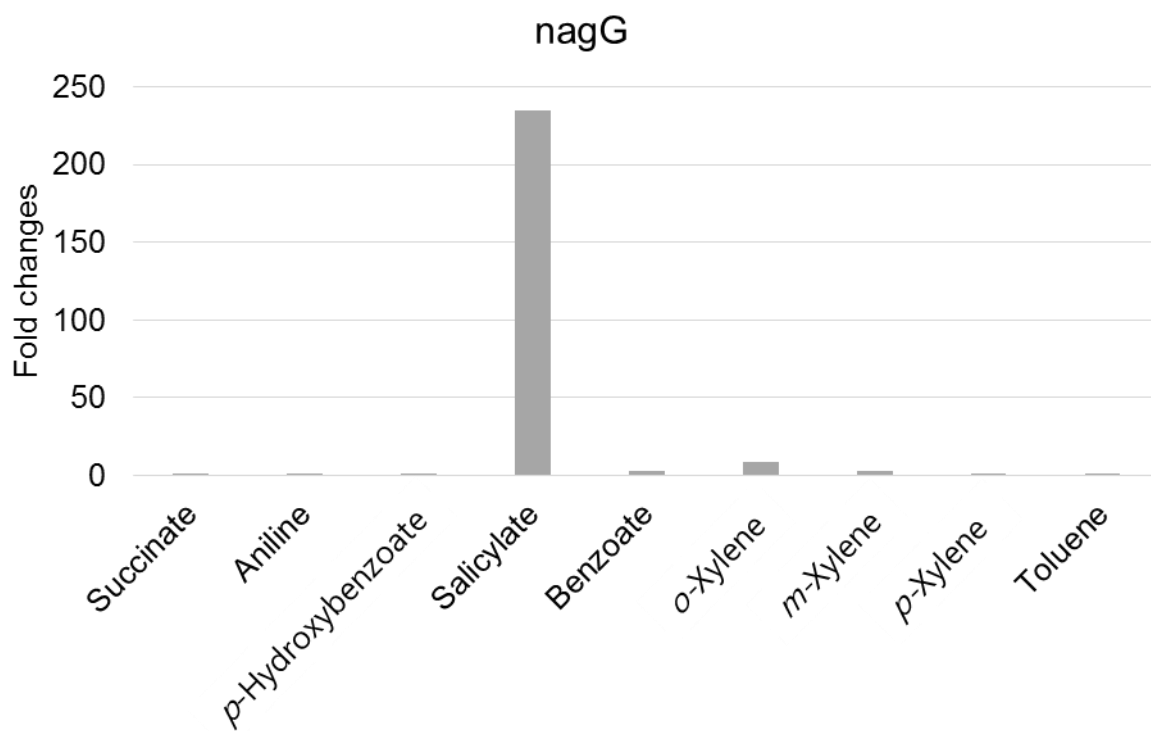

Supplement: S3 Fig — (PDF) [file pone.0154233.s003.pdf]
