# Supplementary material for: Proteogenomic Characterization of Monocyclic Aromatic Hydrocarbon Degradation Pathways in the Aniline-Degrading Bacterium Burkholderia sp. K24
Source: PLoS One. 2016 Apr 28;11(4):e0154233. doi: 10.1371/journal.pone.0154233 (PMC4849787; doi:10.1371/journal.pone.0154233)
Supplement: S1 Table — (DOCX) [file pone.0154233.s004.docx]

**S1 Table.** RT-PCR primers of *Burkholderia* sp. K24

| Locus tag | Gene Name | Product | Primer name | Sequence |
| --- | --- | --- | --- | --- |
| KBK24_0116345 | *catA_1_* | catechol 1,2-dioxygenase | K24_0116345-F | 5'-GCTTTCCGATCTGTTCAAGG-3' |
|  |  |  | K24_0116345-R | 5'-CGATACCCTCAGCTTTGTCC-3' |
| KBK24_0135040 | *catA_2_* | catechol 1,2-dioxygenase | K24_0135040-F | 5'-AAGTCGTCCCACAGATACGG-3' |
|  |  |  | K24_0135040-R | 5'-CGAAGGCAAGTACAGCTTCC-3' |
| KBK24_0125685 | *pcaG* | protocatechuate 3,4-dioxygenase, α subunit | K24_0125685-F | 5'-TGCAATGCTGGAAATCTCAC-3' |
|  |  |  | K24_0125685-R | 5'-GTCACGACCACGTTCAGATG-3' |
| KBK24_0125680 | *pcaH* | protocatechuate 3,4-dioxygenase, β subunit | K24_0125680-F | 5'-TGCACAAGAACGATCAGCAC-3' |
|  |  |  | K24_0125680-R | 5'-GATCGTCAGAAAGCGGTAGC-3' |
| KBK24_0120770 | *boxB* | benzoyl-CoA 2,3-dioxygenase component B | K24_0120770-F | 5'-AGTTCCAGTTGTCGGCACTC-3' |
|  |  |  | K24_0120770-R | 5'-ACATCGTCCGTACCCAGTTC-3' |
| KBK24_0119990 | *dmpB* | catechol 2,3-dioxygenase | K24_0119990-F | 5'-ACCTATACGCGCAGAAGGAA-3' |
|  |  |  | K24_0119990-R | 5'-CAAGAATCAGGCAATGGTCA-3' |
| KBK24_0120285 | nagI | gentisate 1,2-dioxygenase | K24_0120285-F | 5'-GGATTCGCTGAGAAGAGCAG-3' |
|  |  |  | K24_0120285-R | 5'-TGTATCCGAAATGAGCATCG-3' |
| KBK24_0120295 | nagG | salicylate 5-hydroxylase large subunit | K24_0120295-F | 5'-TGTATTGCTGGTTCGAGACG-3' |
|  |  |  | K24_0120295-R | 5'-CACCGTTGACCTTTCCATCT-3' |
