# Supplementary material for: Proteogenomic Characterization of Monocyclic Aromatic Hydrocarbon Degradation Pathways in the Aniline-Degrading Bacterium Burkholderia sp. K24
Source: PLoS One. 2016 Apr 28;11(4):e0154233. doi: 10.1371/journal.pone.0154233 (PMC4849787; doi:10.1371/journal.pone.0154233)
Supplement: S2 Table — (DOCX) [file pone.0154233.s005.docx]

**S2 Table.** Homology analysis of the monocyclic aromatic hydrocarbon degradation genes of *Burkholderia* sp. K24

| Substrates | Locus tag | Gene name | Homologous genes (Identity) | | |
| --- | --- | --- | --- | --- | --- |
| Aniline | KBK24_0116295 | tdnQ | *Frateuria* sp. ANA-18 (99%) | *Delftia tsuruhatensis* strain AD9 (81%) | *Pseudomonas putida* UCC22 (81%) |
|  | KBK24_0116305 | tdnA_1_ | *Frateuria* sp. ANA-18 (99%) | *Delftia* sp. AN3 (79%) | *Delftia acidovorans* (79%) |
|  | KBK24_0116306 | tdnA_2_ | *Frateuria* sp. ANA-18 (99%) | - | - |
|  | KBK24_0116315 | tdnB | *Frateuria* sp. ANA-18 (97%) | *Pseudomonas putida* UCC22 (76%) | *Delftia tsuruhatensis* strain AD9 (76%) |
|  | KBK24_0116296 | tdnT | *Frateuria* sp. ANA-18 (99%) | - | - |
|  | KBK24_0116345 | catA_1_ | *Frateuria* sp. ANA-18 (96%) | *Burkholderia cepacia* strain FB2 (84%) | *Burkholderia contaminans* strain MS14 (82%) |
|  | KBK24_0116335 | catB_1_ | *Frateuria* sp. ANA-18 (99%) | *Burkholderia* sp. CCGE1002 (84%) | *Burkholderia ambifaria* AMMD (83%) |
|  | KBK24_0116340 | catC_1_ | *Frateuria* sp. ANA-18 (100%) | - | - |
|  | KBK24_0135040 | catA_2_ | *Burkholderia fungorum* strain ATCC BAA-463 (99%) | *Frateuria* sp. ANA-18 (99%) | *Burkholderia xenovorans* LB400 (90%) |
|  | KBK24_0135045 | catB_2_ | *Burkholderia fungorum* strain ATCC BAA-463 (99%) | *Frateuria* sp. ANA-18 (98%) | *Burkholderia xenovorans* LB400 (87%) |
|  | KBK24_0135035 | catC_2_ | *Burkholderia fungorum* strain ATCC BAA-463 (99%) | *Frateuria* sp. ANA-18 (98%) | *Burkholderia xenovorans* LB400 (90%) |
|  | KBK24_0116350 | catD | *Frateuria* sp. ANA-18 (98%) | - | - |
| Aniline and  *p*-Hydroxy  benzoate | KBK24_0138210 | pcaI | *Burkholderia fungorum* strain ATCC BAA-463 (98%) | *Burkholderia* sp. CCGE1002 (90%) | *Burkholderia phytofirmans* PsJN (89%) |
|  | KBK24_0138205 | pcaJ | *Burkholderia fungorum* strain ATCC BAA-463 (99%) | *Burkholderia phytofirmans* PsJN (90%) | *Burkholderia* sp. HB1 (90%) |
|  | KBK24_0109905 | pcaF | *Burkholderia fungorum* strain ATCC BAA-463 (100%) | *Burkholderia* sp. CCGE1002 (89%) | *Burkholderia* sp. CCGE1003 (89%) |
| *p*-Hydroxybenzoate | KBK24_0131110 | pobA | *Burkholderia fungorum* strain ATCC BAA-463 (99%) | *Burkholderia phytofirmans* PsJN (87%) | *Burkholderia* sp. CCGE1001 (87%) |
|  | KBK24_0125685 | pcaG | *Burkholderia fungorum* strain ATCC BAA-463 (98%) | *Burkholderia phenoliruptrix* BR3459a (84%) | *Burkholderia xenovorans* LB400 (84%) |
|  | KBK24_0125680 | pcaH | *Burkholderia fungorum* strain ATCC BAA-463 (99%) | *Burkholderia phytofirmans* PsJN (91%) | *Burkholderia xenovorans* LB400 (90%) |
|  | KBK24_0138200 | pcaB | *Burkholderia fungorum* strain ATCC BAA-463 (99%) | *Burkholderia phytofirmans* PsJN (89%) | *Burkholderia xenovorans* LB400 (88%) |
|  | KBK24_0138190 | pcaC | *Burkholderia fungorum* strain ATCC BAA-463 (99%) | *Burkholderia xenovorans* LB400 (91%) | *Burkholderia phytofirmans* PsJN (90%) |
|  | KBK24_0138195 | pcaD | *Burkholderia fungorum* strain ATCC BAA-463 (99%) | *Burkholderia xenovorans* LB400 (89%) | *Burkholderia phytofirmans* PsJN (89%) |
| Benzoate | KBK24_0120745 | badA | *Burkholderia fungorum* strain ATCC BAA-463 (98%) | *Burkholderia xenovorans* LB400 (85%) | *Burkholderia phytofirmans* PsJN (84%) |
|  | KBK24_0120775 | boxA | *Burkholderia fungorum* strain ATCC BAA-463 (99%) | *Burkholderia phytofirmans* PsJN (87%) | *Burkholderia xenovorans* LB400 (86%) |
|  | KBK24_0120770 | boxB | *Burkholderia fungorum* strain ATCC BAA-463 (99%) | *Burkholderia* sp. HB1 (90%) | *Burkholderia* sp. CCGE1002 (90%) |
|  | KBK24_0120765 | boxC | *Burkholderia fungorum* strain ATCC BAA-463 (99%) | *Burkholderia xenovorans* LB400 (89%) | *Burkholderia phytofirmans* PsJN (89%) |
|  | KBK24_0120750 | boxD | *Burkholderia fungorum* strain ATCC BAA-463 (99%) | *Burkholderia xenovorans* LB400 (84%) | *Burkholderia phytofirmans* PsJN (83%) |
|  | KBK24_0113935 | paaF | *Burkholderia fungorum* strain ATCC BAA-463 (99%) | *Burkholderia xenovorans* LB400 (88%) | *Burkholderia phytofirmans* PsJN (88%) |
|  | KBK24_0120670 | paaH | *Burkholderia fungorum* strain ATCC BAA-463 (99%) | *Burkholderia phytofirmans* PsJN (86%) | *Burkholderia xenovorans* LB400 (86%) |
|  | KBK24_0109905 | pcaF | *Burkholderia fungorum* strain ATCC BAA-463 (100%) | *Burkholderia* sp. CCGE1002 (89%) | *Burkholderia* sp. HB1 (89%) |
| Toluene and  *o*-,*m*-,*p*-Xylene | KBK24_0119990 | dmpB | *Burkholderia* sp. HB1 (100%) | *Burkholderia* sp. RP007 (99%) | - |
|  | KBK24_0119995 | dmpC | *Burkholderia* sp. HB1 (99%) | *Burkholderia* sp. RP007 (98%) | - |
|  | KBK24_0120030 | dmpD | *Burkholderia* sp. HB1 (99%) | - | - |
|  | KBK24_0120020 | dmpI | *Burkholderia* sp. HB1 (100%) | - | - |
|  | KBK24_0120015 | dmpH | *Burkholderia* sp. HB1 (99%) | - | - |
|  | KBK24_0120000 | dmpE | *Burkholderia* sp. HB1 (99%) | - | - |
|  | KBK24_0120010 | dmpG | *Burkholderia* sp. HB1 (99%) | *Pseudomonas* sp. S-47 (78%) | *Pseudomonas* sp. KB35B (77%) |
|  | KBK24_0120005 | dmpF | *Burkholderia* sp. HB1 (99%) | *Thauera* sp. MZ1T (77%) | *Pseudomonas* sp. OX1 (77%) |
| Salicylate | KBK24_0120295 | nagG | *Burkholderia* sp. HB1 (99%) | *Burkholderia* sp. C3 (98%) | *Burkholderia fungorum* strain ATCC BAA-463 (97%) |
|  | KBK24_0120300 | nagH | *Burkholderia* sp. HB1 (98%) | *Burkholderia fungorum* strain ATCC BAA-463 (98%) | *Burkholderia* sp. C3 (97%) |
|  | KBK24_0120285 | nagI | *Burkholderia* sp. HB1 (100%) | *Burkholderia fungorum* strain ATCC BAA-463 (99%) | *Burkholderia* sp. C3 (97%) |
|  | KBK24_0120310 | nagL | *Burkholderia* sp. HB1 (99%) | *Burkholderia fungorum* strain ATCC BAA-463 (98%) | - |
|  | KBK24_0120290 | nagK | *Burkholderia* sp. HB1 (99%) | *Burkholderia fungorum* strain ATCC BAA-463 (99%) | *Burkholderia* sp. C3 (97%) |
